# 2-{[3-Chloro-4-(4-chloro­phen­oxy)phen­yl]imino­meth­yl}-4-nitro­phenol

**DOI:** 10.1107/S1600536813012518

**Published:** 2013-05-15

**Authors:** Nevzat Karadayı, Yavuz Köysal, Songül Şahin, Emine Coşkun, Orhan Büyükgüngör

**Affiliations:** a Yeşilyurt Demir Celik Higher Vocational School, Ondokuz Mayıs University, TR-55300 Samsun, Turkey; b Department of Chemistry, Faculty of Arts and Sciences, Ondokuz Mayıs University, TR-55139 Samsun, Turkey; c Department of Physics, Faculty of Arts and Sciences, Ondokuz Mayıs University, TR-55139 Samsun, Turkey

## Abstract

In the title compound, C_19_H_12_Cl_2_N_2_O_4_, the imine bond length of 1.257 (6) Å is typical of a double bond. The dihedral angle between the *para*-nitro benzene ring and the central benzene ring is 12.06 (3)° and that between the central benzene and the *para*-chloro benzene ring is 73.81 (2)°. An intra­molecular O—H⋯N hydrogen bond generates an *S*(6) ring motif. In the crystal, mol­ecules are linked together by two pairs of C—H⋯O interactions (to the same O atom acceptor), forming inversion dimers. A short Cl⋯Cl contact [3.232 (4) Å] is observed.

## Related literature

For applications of related Schiff base compounds, see: Santos *et al.* (2001 ▶); Cohen *et al.* (1964 ▶). For related structures, see: Aygün *et al.* (1998 ▶); Karadayı *et al.* (2003 ▶, 2005 ▶, 2006 ▶); Faridbod *et al.* (2008 ▶); Raja *et al.* (2008 ▶); Li & Zhang (2004 ▶); Köysal *et al.* (2012 ▶). For standard bond lengths, see: Allen *et al.* (1987 ▶).
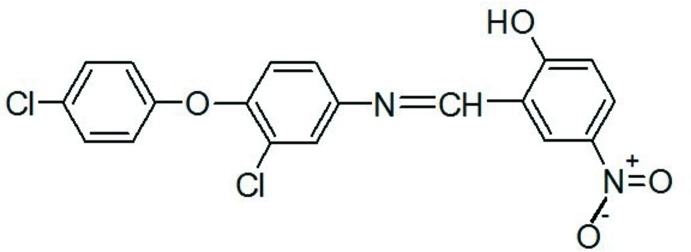


## Experimental

### 

#### Crystal data

C_19_H_12_Cl_2_N_2_O_4_
*M* *_r_* = 403.21
Triclinic, 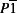

*a* = 5.5649 (8) Å
*b* = 7.929 (1) Å
*c* = 21.778 (4) Å
α = 86.260 (12)°
β = 83.596 (12)°
γ = 70.739 (10)°
*V* = 901.1 (2) Å^3^
*Z* = 2
Mo *K*α radiation
μ = 0.39 mm^−1^
*T* = 296 K
0.76 × 0.39 × 0.03 mm

#### Data collection

Stoe IPDS 2 diffractometer
Absorption correction: integration (*X-RED32*; Stoe & Cie, 2002 ▶) *T*_min_ = 0.849, *T*_max_ = 0.985
10061 measured reflections
3475 independent reflections
1473 reflections with *I* > 2σ(*I*)
*R*_int_ = 0.076

#### Refinement

*R*[*F* ^2^ > 2σ(*F* ^2^)] = 0.068
*wR*(*F* ^2^) = 0.186
*S* = 1.05
3475 reflections
248 parameters
52 restraints
H-atom parameters constrained
Δρ_max_ = 0.20 e Å^−3^
Δρ_min_ = −0.22 e Å^−3^

### 

Data collection: *X-AREA* (Stoe & Cie, 2002 ▶); cell refinement: *X-AREA*; data reduction: *X-RED32* (Stoe & Cie, 2002 ▶); program(s) used to solve structure: *SHELXS97* (Sheldrick, 2008 ▶); program(s) used to refine structure: *SHELXL97* (Sheldrick, 2008 ▶); molecular graphics: *ORTEP-3 for Windows* (Farrugia, 2012 ▶); software used to prepare material for publication: *WinGX* (Farrugia, 2012 ▶).

## Supplementary Material

Crystal structure: contains datablock(s) global, I. DOI: 10.1107/S1600536813012518/rn2114sup1.cif

Structure factors: contains datablock(s) I. DOI: 10.1107/S1600536813012518/rn2114Isup2.hkl

Supplementary material file. DOI: 10.1107/S1600536813012518/rn2114Isup3.cml

Additional supplementary materials:  crystallographic information; 3D view; checkCIF report

## Figures and Tables

**Table 1:** Hydrogen-bond geometry (Å, °)

*D*—H⋯*A*	*D*—H	H⋯*A*	*D*⋯*A*	*D*—H⋯*A*
O1—H1⋯N1	0.82	1.85	2.564 (5)	145
C7—H7⋯O3^i^	0.93	2.51	3.321 (6)	146
C6—H6⋯O3^i^	0.93	2.49	3.319 (4)	148

Symmetry code: (i) 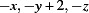.

**Fig. 1.**
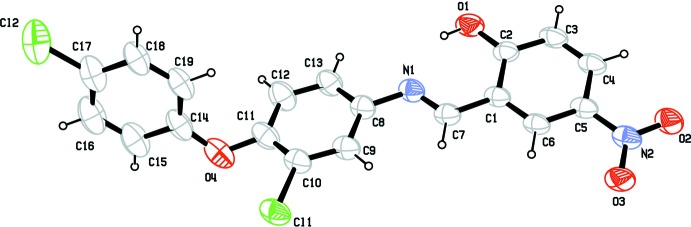
An drawing of the title compound showing the atomic numbering scheme. Displacement ellipsoids of non-H atoms are shown at the 30% probability level.

**Fig. 2.**
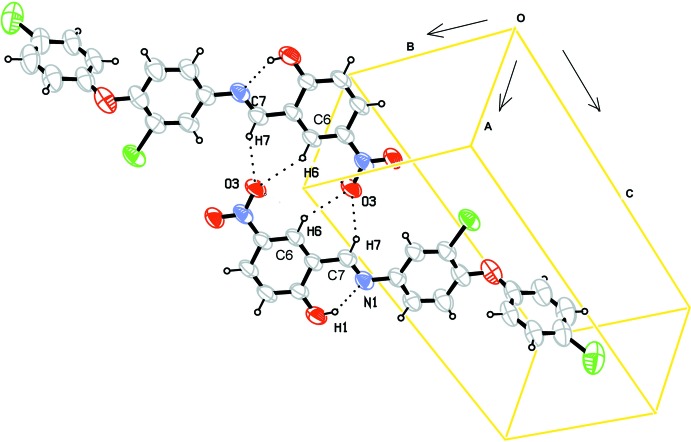
A view of the hydrogen-bonding network in the title compound. Hydrogen bonds are indicated by dashed line.

## supplementary crystallographic information

### Comment

Schiff base derivatives are found to exhibit important pharmacological properties, such as antibacterial, antitumor and antitoxic activities (Santos *et al.*, 2001). Schiff base compounds can be classified by their photochromic and thermochromic characteristics (Cohen *et al.*, 1964). In this study we report the structure of (I). The molecule structure of (I) is shown in Fig.1.

The N1=C7 bond length is 1.257 (6) Å, approximately equal to previously reported C=N double-bond lengths (Allen *et al.*, 1987; Aygün *et al.*, 1998; Karadayı *et al.*, 2003; Faridbod *et al.*, 2008). The geometric parameters in (I) are comparable with the similar reported structures (Raja *et al.*, 2008; Li & Zhang, 2004; Karadayı *et al.*, 2005; Karadayı *et al.*, 2006; Köysal *et al.*, 2012). The dihedral angles between the aromatic rings (C1—C6) and (C8—C13) is 12.06 (3)° and (C8—C13) and (C14—C19) is 73.81 (2)°.

The H atom is located on the hydroxy O atom rather than on the N atom. The molecular intramolecular O—H···N hydrogen bond result in formation of six-membered ring and generates an S(6) ring motif. The molecules in crystal are held together by two intermolecular C—H···O hydrogen bonds. (Table 1; Fig. 2).

### Experimental

The compound 2-{[3-chloro-4-(4-chlorophenoxy)phenyl]carbonoimidoyl}-4-nitrophenol was prepared by reflux a mixture of a solution containing 2-hydroxy-5-nitrobenzaldehyde(0.014 g, 0.082 mmol) and a solution containing 3-Chloro-4-(4-chlorophenoxy)aniline(0,021 g, 0.082 mmol) in 20 ml e thanol. The reaction mixture was stirred for 1 h under reflux. The crystals of suitable for 2-{[3-chloro-4-(4-chlorophenoxy)phenyl]carbonoimidoyl}-4-nitrophenol X-ray analysis were obtained from ethylalcohol by slow evaporation (yield 58%). Mp: 436–438 K. Elemental analysis: Uv-vis (CHCl_3_): λ=212 (A: 2,253), 242 (A: 2,186), 304 nm. (A: 1,019). IR (ν_max_, cm^-1^): 3090–3030(Ar—CH_2_), 2840–2940 (CH_2_), 2210 (CN), 1621, 1597, 1570, 1519, 1481, 1348, 1298, 1284, 1259, 1212, 1184, 1162, 1130, 1101, 1090, 1051, 1010, 976, 942, 927, 912, 870, 836, 823,780, 754, 725, 640, 598, 576, 546, 495.

### Refinement

All H atoms were placed in calculated positions and refined using a riding model, with fixed C—H distances of 0.93 Å and an O—H distance of 0.82 Å. The isotropic displacement parameters of the H atoms were fixed at 1.2*U*_eq_ (C—H) and 1.5*U*_eq_ (O—H) of their parents atoms.

### Crystal data

**Table:**

C_19_H_12_Cl_2_N_2_O_4_	*Z* = 2
*M**_r_* = 403.21	*F*(000) = 412
Triclinic, *P*1	*D*_x_ = 1.486 Mg m^−^^3^
Hall symbol: -P 1	Mo *K*α radiation, λ = 0.71073 Å
*a* = 5.5649 (8) Å	Cell parameters from 8340 reflections
*b* = 7.929 (1) Å	θ = 1.9–28.2°
*c* = 21.778 (4) Å	µ = 0.39 mm^−^^1^
α = 86.260 (12)°	*T* = 296 K
β = 83.596 (12)°	Plate, yellow
γ = 70.739 (10)°	0.76 × 0.39 × 0.03 mm
*V* = 901.1 (2) Å^3^	

### Data collection

**Table:**

Stoe IPDS 2 diffractometer	3475 independent reflections
Radiation source: fine-focus sealed tube	1473 reflections with *I* > 2σ(*I*)
Graphite monochromator	*R*_int_ = 0.076
ω scans	θ_max_ = 26.0°, θ_min_ = 1.9°
Absorption correction: integration (*X-RED32*; Stoe & Cie, 2002)	*h* = −6→6
*T*_min_ = 0.849, *T*_max_ = 0.985	*k* = −9→9
10061 measured reflections	*l* = −26→26

### Refinement

**Table:**

Refinement on *F*^2^	Secondary atom site location: difference Fourier map
Least-squares matrix: full	Hydrogen site location: inferred from neighbouring sites
*R*[*F*^2^ > 2σ(*F*^2^)] = 0.068	H-atom parameters constrained
*wR*(*F*^2^) = 0.186	*w* = 1/[σ^2^(*F*_o_^2^) + (0.0634*P*)^2^ + 0.1371*P*] where *P* = (*F*_o_^2^ + 2*F*_c_^2^)/3
*S* = 1.05	(Δ/σ)_max_ < 0.001
3475 reflections	Δρ_max_ = 0.20 e Å^−^^3^
248 parameters	Δρ_min_ = −0.22 e Å^−^^3^
52 restraints	Extinction correction: *SHELXL97* (Sheldrick, 2008), Fc^*^=kFc[1+0.001xFc^2^λ^3^/sin(2θ)]^-1/4^
Primary atom site location: structure-invariant direct methods	Extinction coefficient: 0.011 (3)

### Special details

**Table:**

Geometry. All e.s.d.'s (except the e.s.d. in the dihedral angle between two l.s. planes) are estimated using the full covariance matrix. The cell e.s.d.'s are taken into account individually in the estimation of e.s.d.'s in distances, angles and torsion angles; correlations between e.s.d.'s in cell parameters are only used when they are defined by crystal symmetry. An approximate (isotropic) treatment of cell e.s.d.'s is used for estimating e.s.d.'s involving l.s. planes.
Refinement. Refinement of *F*^2^ against ALL reflections. The weighted *R*-factor *wR* and goodness of fit *S* are based on *F*^2^, conventional *R*-factors *R* are based on *F*, with *F* set to zero for negative *F*^2^. The threshold expression of *F*^2^ > σ(*F*^2^) is used only for calculating *R*-factors(gt) *etc*. and is not relevant to the choice of reflections for refinement. *R*-factors based on *F*^2^ are statistically about twice as large as those based on *F*, and *R*- factors based on ALL data will be even larger.

### Fractional atomic coordinates and isotropic or equivalent isotropic displacement parameters (Å^2^)

**Table:**

	*x*	*y*	*z*	*U*_iso_*/*U*_eq_	
Cl2	0.7962 (6)	−0.3765 (3)	0.45465 (11)	0.2198 (11)	
Cl1	−0.0539 (2)	0.30360 (16)	0.22683 (8)	0.1373 (6)	
C18	0.6896 (14)	−0.0931 (8)	0.3740 (3)	0.144 (2)	
H18	0.8584	−0.1407	0.3573	0.173*	
C17	0.6001 (16)	−0.1794 (9)	0.4229 (3)	0.156 (2)	
C19	0.5349 (14)	0.0624 (8)	0.3493 (3)	0.142 (2)	
H19	0.5998	0.1220	0.3168	0.171*	
C16	0.3486 (18)	−0.1156 (11)	0.4467 (3)	0.177 (3)	
H16	0.2860	−0.1763	0.4792	0.213*	
O3	0.0233 (6)	1.2311 (4)	−0.04140 (18)	0.1345 (13)	
C15	0.1920 (16)	0.0391 (11)	0.4217 (3)	0.161 (3)	
H15	0.0222	0.0837	0.4379	0.193*	
C14	0.2795 (15)	0.1313 (9)	0.3728 (3)	0.135 (2)	
O2	0.2457 (6)	1.4083 (4)	−0.05235 (18)	0.1303 (13)	
N2	0.1826 (7)	1.2896 (5)	−0.0236 (3)	0.1123 (14)	
C10	0.1116 (9)	0.4319 (6)	0.2530 (3)	0.1125 (17)	
C6	0.2397 (8)	1.0768 (5)	0.0639 (3)	0.1007 (16)	
H6	0.1264	1.0298	0.0487	0.121*	
C9	0.1661 (8)	0.5615 (5)	0.2153 (3)	0.1149 (17)	
H9	0.1109	0.5833	0.1759	0.138*	
C5	0.2973 (8)	1.2175 (5)	0.0331 (3)	0.1021 (16)	
O4	0.1060 (9)	0.2799 (7)	0.3499 (2)	0.1582 (18)	
C11	0.1866 (11)	0.3996 (7)	0.3121 (3)	0.129 (2)	
C4	0.4639 (9)	1.2908 (6)	0.0547 (3)	0.1136 (18)	
H4	0.5006	1.3863	0.0335	0.136*	
C1	0.3481 (8)	1.0065 (5)	0.1168 (3)	0.0988 (15)	
C7	0.2800 (9)	0.8626 (6)	0.1508 (3)	0.1049 (16)	
H7	0.1647	0.8174	0.1355	0.126*	
C8	0.3057 (8)	0.6620 (6)	0.2360 (3)	0.1062 (15)	
C2	0.5222 (9)	1.0794 (6)	0.1393 (3)	0.1050 (15)	
N1	0.3733 (7)	0.7978 (5)	0.2004 (2)	0.1090 (13)	
C3	0.5729 (9)	1.2220 (7)	0.1071 (3)	0.1227 (19)	
H3	0.6844	1.2715	0.1219	0.147*	
O1	0.6321 (7)	1.0140 (5)	0.1906 (2)	0.1368 (13)	
H1	0.6007	0.9221	0.2019	0.205*	
C13	0.3831 (11)	0.6245 (7)	0.2947 (3)	0.1260 (18)	
H13	0.4780	0.6887	0.3087	0.151*	
C12	0.3265 (11)	0.4975 (8)	0.3331 (3)	0.147 (2)	
H12	0.3801	0.4765	0.3727	0.177*	

### Atomic displacement parameters (Å^2^)

**Table:**

	*U*^11^	*U*^22^	*U*^33^	*U*^12^	*U*^13^	*U*^23^
Cl2	0.339 (3)	0.1660 (17)	0.1594 (17)	−0.100 (2)	−0.0038 (18)	0.0157 (15)
Cl1	0.1270 (10)	0.1025 (8)	0.1993 (15)	−0.0557 (7)	−0.0076 (9)	−0.0410 (9)
C18	0.203 (6)	0.128 (4)	0.126 (5)	−0.095 (4)	0.022 (4)	−0.023 (4)
C17	0.258 (7)	0.142 (5)	0.100 (4)	−0.112 (5)	0.006 (5)	−0.019 (3)
C19	0.190 (6)	0.125 (4)	0.140 (5)	−0.098 (4)	0.020 (4)	−0.021 (4)
C16	0.273 (9)	0.182 (6)	0.103 (5)	−0.124 (6)	0.038 (5)	−0.013 (4)
O3	0.137 (2)	0.103 (2)	0.195 (4)	−0.068 (2)	−0.055 (2)	−0.010 (2)
C15	0.229 (7)	0.175 (5)	0.106 (5)	−0.113 (5)	0.033 (5)	−0.025 (4)
C14	0.196 (6)	0.123 (4)	0.120 (4)	−0.100 (4)	0.019 (5)	−0.037 (4)
O2	0.134 (3)	0.0930 (19)	0.184 (4)	−0.0598 (19)	−0.028 (2)	−0.004 (2)
N2	0.098 (3)	0.071 (2)	0.178 (4)	−0.037 (2)	−0.009 (3)	−0.032 (3)
C10	0.104 (3)	0.080 (3)	0.157 (5)	−0.032 (2)	0.000 (3)	−0.037 (3)
C6	0.081 (3)	0.069 (2)	0.160 (5)	−0.030 (2)	−0.014 (3)	−0.024 (3)
C9	0.106 (3)	0.080 (2)	0.164 (5)	−0.031 (2)	−0.016 (3)	−0.033 (3)
C5	0.081 (3)	0.070 (2)	0.161 (5)	−0.027 (2)	−0.008 (3)	−0.028 (3)
O4	0.169 (4)	0.153 (3)	0.178 (4)	−0.095 (3)	0.025 (3)	−0.029 (4)
C11	0.139 (4)	0.110 (4)	0.154 (5)	−0.065 (3)	0.017 (4)	−0.034 (4)
C4	0.095 (3)	0.086 (3)	0.177 (5)	−0.048 (3)	−0.018 (3)	−0.023 (3)
C1	0.079 (3)	0.066 (2)	0.157 (5)	−0.026 (2)	−0.013 (3)	−0.023 (3)
C7	0.090 (3)	0.077 (3)	0.153 (5)	−0.027 (2)	−0.017 (3)	−0.024 (3)
C8	0.104 (3)	0.079 (3)	0.139 (5)	−0.032 (2)	−0.009 (3)	−0.022 (3)
C2	0.085 (3)	0.086 (3)	0.151 (5)	−0.031 (2)	−0.027 (3)	−0.013 (3)
N1	0.103 (3)	0.080 (2)	0.153 (4)	−0.037 (2)	−0.015 (3)	−0.021 (3)
C3	0.098 (3)	0.106 (3)	0.186 (6)	−0.057 (3)	−0.024 (4)	−0.018 (4)
O1	0.120 (3)	0.124 (3)	0.191 (4)	−0.063 (2)	−0.045 (3)	−0.004 (3)
C13	0.145 (4)	0.111 (4)	0.143 (5)	−0.066 (3)	−0.016 (4)	−0.019 (4)
C12	0.189 (6)	0.141 (4)	0.143 (5)	−0.094 (5)	−0.009 (4)	−0.023 (4)

### Geometric parameters (Å, º)

**Table:**

Cl2—C17	1.735 (8)	C9—C8	1.405 (6)
Cl1—C10	1.737 (4)	C9—H9	0.9300
C18—C17	1.365 (8)	C5—C4	1.381 (6)
C18—C19	1.367 (8)	O4—C11	1.370 (7)
C18—H18	0.9300	C11—C12	1.394 (7)
C17—C16	1.374 (9)	C4—C3	1.350 (7)
C19—C14	1.394 (8)	C4—H4	0.9300
C19—H19	0.9300	C1—C2	1.424 (6)
C16—C15	1.370 (10)	C1—C7	1.452 (7)
C16—H16	0.9300	C7—N1	1.257 (6)
O3—N2	1.234 (4)	C7—H7	0.9300
C15—C14	1.386 (8)	C8—C13	1.377 (7)
C15—H15	0.9300	C8—N1	1.412 (6)
C14—O4	1.360 (8)	C2—O1	1.326 (6)
O2—N2	1.222 (5)	C2—C3	1.383 (7)
N2—C5	1.449 (6)	C3—H3	0.9300
C10—C9	1.365 (7)	O1—H1	0.8200
C10—C11	1.379 (8)	C13—C12	1.361 (7)
C6—C1	1.360 (6)	C13—H13	0.9300
C6—C5	1.375 (6)	C12—H12	0.9300
C6—H6	0.9300		
			
C17—C18—C19	121.0 (7)	C4—C5—N2	119.3 (5)
C17—C18—H18	119.5	C14—O4—C11	120.2 (5)
C19—C18—H18	119.5	O4—C11—C10	118.5 (5)
C18—C17—C16	120.5 (8)	O4—C11—C12	121.8 (7)
C18—C17—Cl2	121.1 (7)	C10—C11—C12	119.5 (6)
C16—C17—Cl2	118.3 (6)	C3—C4—C5	119.2 (5)
C18—C19—C14	119.8 (6)	C3—C4—H4	120.4
C18—C19—H19	120.1	C5—C4—H4	120.4
C14—C19—H19	120.1	C6—C1—C2	119.3 (5)
C15—C16—C17	118.7 (7)	C6—C1—C7	120.2 (4)
C15—C16—H16	120.6	C2—C1—C7	120.5 (6)
C17—C16—H16	120.6	N1—C7—C1	121.4 (5)
C16—C15—C14	121.9 (8)	N1—C7—H7	119.3
C16—C15—H15	119.1	C1—C7—H7	119.3
C14—C15—H15	119.1	C13—C8—C9	117.8 (5)
O4—C14—C15	117.2 (7)	C13—C8—N1	117.9 (5)
O4—C14—C19	124.7 (6)	C9—C8—N1	124.3 (6)
C15—C14—C19	118.1 (8)	O1—C2—C3	120.2 (5)
O2—N2—O3	121.7 (5)	O1—C2—C1	121.0 (5)
O2—N2—C5	118.7 (4)	C3—C2—C1	118.7 (6)
O3—N2—C5	119.6 (4)	C7—N1—C8	123.2 (5)
C9—C10—C11	121.1 (5)	C4—C3—C2	121.4 (5)
C9—C10—Cl1	120.1 (5)	C4—C3—H3	119.3
C11—C10—Cl1	118.8 (5)	C2—C3—H3	119.3
C1—C6—C5	120.0 (4)	C2—O1—H1	109.5
C1—C6—H6	120.0	C12—C13—C8	122.8 (5)
C5—C6—H6	120.0	C12—C13—H13	118.6
C10—C9—C8	120.0 (6)	C8—C13—H13	118.6
C10—C9—H9	120.0	C13—C12—C11	118.8 (6)
C8—C9—H9	120.0	C13—C12—H12	120.6
C6—C5—C4	121.3 (6)	C11—C12—H12	120.6
C6—C5—N2	119.3 (4)		
			
C14—C15—C16—C17	0.8 (11)	C16—C17—C18—C19	2.7 (9)
C15—C16—C17—C18	−2.0 (10)		

### Hydrogen-bond geometry (Å, º)

**Table:**

*D*—H···*A*	*D*—H	H···*A*	*D*···*A*	*D*—H···*A*
O1—H1···N1	0.82	1.85	2.564 (5)	145
C7—H7···O3^i^	0.93	2.51	3.321 (6)	146
C6—H6···O3^i^	0.93	2.49	3.319 (4)	148

Symmetry code: (i) −*x*, −*y*+2, −*z*.
